# Weeds

**DOI:** 10.3201/eid1206.AD1206

**Published:** 2006-06

**Authors:** Ronald O. Valdiserri

**Affiliations:** * Centers for Disease Control and Prevention, Atlanta, Georgia, USA

**Keywords:** AIDS, HIV, weeds, another dimension

The even mead, that erst brought sweetly forth
The freckled cowslip, burnet, and green clover,
Wanting the scythe, all uncorrected, rank,
Conceives by idleness, and nothing teems
But hateful docks, rough thistles, kecksies, burs,
Losing both beauty and utility.

William Shakespeare

Henry V

Sometimes in the spring, when I'm on my hands and knees among the azaleas, pulling out purslane and dandelion, I think how wonderful gardening would be if only it weren't for weeds. Most of the time I dislike weeding, especially when I see it as a battle. Given the natural guile of the enemy, I become pessimistic, thinking that the weeds will always be one step ahead of me, that I can never defeat them. I can create huge mounds of compost from the honeysuckle vine, chickweed, and pokeberry that I remove from my woodland garden beds and still hundreds of malignant survivors will have escaped my surveillance. When my frustration grows past endurance, my war escalates to chemical weapons. I don't spray herbicides very often, though, for I'm not proud of resorting to excessive force. Weeds, it seems, are truly like troubles: prodigious, vexing, and capable of bringing out the worst in people. But like troubles, they can also coax out the best.

Sometimes, when I'm weeding, the simple action of plunging the fork into the earth, uprooting the plant, and shaking loose the soil that clings to its roots is very soothing. Plunge, pull, shake—repeated over and over, just like a mantra. On these occasions, my thoughts turn philosophical. Maybe the strong smell of the earth loosens memories from an earlier, less careworn time. Or maybe this contemplative sensibility is a byproduct of repetitious action. In either case, when this mood takes over, I lose the notion that the weeds are my enemy, that their leaves and roots are acting malevolently. The weeds, I realize, are neither good nor bad; they simply exist. Gardens do not grow without weeds, and life does not unfold without misfortune. And then I think about the way my life has been touched by AIDS.

I can still remember the first time I came across a description of an unusual immune disorder in a medical journal—long before the disease even had a name. Because of my training in pathology, I was curious about this mysterious new disease. I remember feeling a ghoulish interest in an illness that could cause such tremendous, irreparable damage. Even then, before any of us knew its pathogenesis, its destructive potential was clear, and I felt, perhaps, much the same as the physicists who first glimpsed the horrendous possibilities of the atomic bomb. My scientific detachment didn't live long; AIDS soon became more than a medical curiosity for me. It began to take people I knew, friends who I had hoped would surround me all my life. Sources of comfort and understanding dried up. I started feeling like an uprooted, disenfranchised farmer of Dust Bowl days, driven from once green and fertile land and forced to migrate to unknown places.

Over that first horrible decade of its debut, AIDS managed to dissolve my sense of permanence and distort my perception of time. Before AIDS, I didn't know much about misfortune. I thought about the future primarily in terms of its potential to bring me more gratification and greater achievements. After AIDS, I understood that the future also has the power to disappoint. That knowledge has made me supremely impatient. Now it seems that nothing is quick enough for me; everything takes too long. I feel as if my internal clock has been wound so tightly that at any moment the hands might go spinning clean off the face, so rapidly are they going around and around.

AIDS changed my professional life as well. I had been content to study disease from a distance, behind a microscope. As a pathologist I thought about illness primarily as a disruption in normal physiology, and I was satisfied with the contributions I could make to patient care by diagnosing unusual tumors and peculiar lesions. But after the epidemic took hold, my interest in abnormal physiology waned. The accurate diagnosis of diseases, including AIDS, though it is an essential step in patient care, had become too passive for me, seemed to imply acceptance of the inevitability of illness. No longer satisfied with identifying disease, I wanted to learn how to prevent it from occurring. I was determined to join those who were already trying to stop the spread of the epidemic. And so, after I finished my public health degree, I left my job as a university-based pathologist to work full-time on AIDS prevention at the Centers for Disease Control and Prevention.

It would be easy to assume that the AIDS epidemic, so often associated with loss, generates nothing but sorrow in those it touches, that the personal adjustments we make are all negative. I find, on the contrary, that AIDS has helped me to clarify just what does and does not matter during our brief time in the world. The epidemic has not taken hope away from me, but it has taught me the inadequacy of looking toward the future as a means of rescue from the present. AIDS has shown me that hope is strongest in us when we seek our fulfillment in the circumstances of the present, when we refuse to defer our dreams or to accept defeat.

Sometimes when I'm working in my garden, I think about the invasiveness of weeds and disease, how they appear in the garden, in the human population, uninvited, unwelcome, capable of causing tremendous destruction if left unattended. Yes, it would be wonderful if some spectacular act of God or nature would banish AIDS from our existence. Gardeners dream that they'll awake some morning to find that the crabgrass has magically departed from the perennial beds. Weeds, though, whether real or metaphorical, will not be banished. Pain and loss are intrinsic to our existence in the temporal world. The best way to keep weeds from engulfing the garden is not to rail at the injustice of fate but to get down on our hands and knees and begin clearing them away.

Reprinted from *Gardening in Clay: Reflections on AIDS*, by Ronald O. Valdiserri. Copyright © 1994 by Cornell University. Used by permission of the publisher, Cornell University Press.

## Author's Note

I wrote "Weeds" in the early 1990s (Figure); the essay appeared in a collection on AIDS, Gardening in Clay (*1*). Part memoir, part social commentary, the book sprung from my desire to honor and eventually memorialize my twin brother Edwin—who sickened and died of AIDS during the course of my writing the book.

**Figure:**
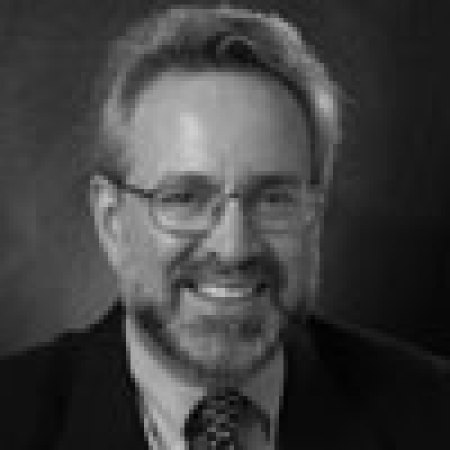
Dr Valdiserri is Deputy Director of the National Center for HIV, STD, and TB Prevention (NCHSTP) at CDC. He is currently working on a text addressing health disparities among gay and bisexual men.

In 1992, the year Edwin died, tennis great Arthur Ashe announced he had AIDS; the Centers for Disease Control and Prevention estimated 140,000–168,000 persons were living with AIDS in the United States (*2*); the International AIDS Society moved its 8th International Conference from Boston to Amsterdam in protest of US policy on HIV-infected travelers; and the Food and Drug Administration was some 3 years off approving the first protease inhibitor, saquinavir.

How far we've come in the 25 years since the first AIDS cases were reported in the United States is difficult to ignore (*3*). Gone is the bleak pessimism that accompanied diagnosis of infection with human T-cell lymphotropic virus type III or lymphadenopathy-associated virus, as the virus was formerly known (*4*). Today, persons who learn they are infected with HIV can enjoy longer and healthier lives as a result of highly active combination antiretroviral therapy. Gone, too, are the urgency and importance Americans once placed on AIDS, despite its continued and substantial national toll, especially in communities of color (*5*).

As "Weeds" suggests, in the continuing chronicles of humans versus retroviruses, we are far from ridding our garden of weeds.
